# Longitudinal retinal functional evaluation using full-field electroretinography in New Zealand white and Dutch Belted rabbits with aging

**DOI:** 10.1038/s41598-025-17856-8

**Published:** 2025-08-29

**Authors:** Van Phuc Nguyen, Yeachan Lee, Zhuying Wei, Mi Zheng, Khoi Tran, Graham Lippincott, Wei Zheng, Y. Eugene Chen, Dongshan Yang, Yannis M. Paulus

**Affiliations:** 1 Department of Ophthalmology, Wilmer Eye Institute, Johns Hopkins University, Baltimore, MD 21287 USA; 2 Department of Ophthalmology and Visual Sciences, University of Michigan, Ann Arbor, MI 48105 USA; 3 Center for Advanced Models for Translational Sciences and Therapeutics, Department of Internal Medicine, University of Michigan, 2800 Plymouth Rd, Ann Arbor, MI 48109-2800 USA; 4 Department of Biomedical Engineering, Johns Hopkins University, Baltimore, MD 21287 USA; 5 Department of Ophthalmology, Fujian Provincial Hospital, Fujian, 350001 China; 6 Department of Ophthalmology, Jiangnan University Medical Center, Wuxi, 214002 China; 7 Department of Ophthalmology, Department of Biomedical Engineering, Johns Hopkins University, 600 N. Wolfe Street, Baltimore, MD 21287 USA

**Keywords:** Computational science, Information technology, Scientific data

## Abstract

Electroretinography (ERG) is pivotal in elucidating retinal function, yet investigations into the temporal dynamics of ERG signals in New Zealand White (NZW) and Dutch-belted (DB) rabbits remain scarce. This study presents a longitudinal assessment of retinal function in both NZW and DB strains. ERG recordings were conducted on four NZW and four DB rabbits at 2, 7, 15, and 24 months of age, encompassing both dark-adapted and light-adapted protocols at each time point. Quantitative analyses included assessment of a- and b-wave amplitudes, implicit times, and photopic flicker responses. Results revealed consistently stronger a- and b-wave amplitudes in NZW rabbits compared to DB rabbits across all time points. These stronger ERG responses likely result from increased effective light exposure at the photoreceptor level in NZW rabbits, rather than indicating intrinsic differences in retinal sensitivity. Over time, NZW rabbits showed a decline in visual function of the cone and postreceptoral systems, with the rod system less affected. In contrast, the visual function of DB rabbits initially improved at an early stage, followed by a slight decline after 15 months. The differences between the two strains may be attributed to the varying speeds of retinal maturation, melanin’s absorption of light, and its protective effect against light-induced retinal damage. This dataset underscores the differential retinal characteristics between NZW and DB rabbits, shedding light on their distinct functional profiles.

## Introduction

In recent decades, significant advances have been made in technology that enable objective assessments of retinal structure and function. Among these, full-field electroretinography (ff-ERG) remains one of the most established and widely used techniques for recording the global electrical response of the retina to light stimuli^1^. ERG holds immense value in both clinical and research settings, particularly in animal experiments where objective evaluation of visual acuity is challenging. A number of studies have been conducted on normal ERGs in animals, although the majority of these have focused on rats and mice^2^.

The ERG waveform is composed of several components, each arising from distinct retinal cell populations. The a-wave reflects the initial hyperpolarization of photoreceptors in response to light, indicating outer retinal function, while the b-wave originates primarily from bipolar and Müller cells and represents the subsequent inner retinal response to photoreceptor activation. Oscillatory potentials (OPs), which are high-frequency wavelets superimposed on the ascending limb of the b-wave, are believed to reflect inner retinal activity, particularly from amacrine cells^3,4^. These cellular contributions provide important context for interpreting age- and pigmentation-related changes in ERG signals.

Compared to other experimental animals, rabbits are not only easily handled and maintained, but also possess a longer lifespan, which is ideal for studying age-related diseases. Additionally, rabbits have relatively large eyes, sharing many anatomical and biochemical features with humans, making them particularly suitable for ophthalmic research, especially for the pre-clinical efficacy and safety assessments^5^. In laboratory conditions, rabbits typically reach sexual maturity around 4–6 months of age. They are considered young adults between 6 and 12 months, adults from 1 to 3 years, and geriatric beyond 3–4 years of age^6^. This classification provides a useful framework for interpreting the age- related changes explored in this study.

Despite their suitability, comprehensive studies on the baseline ERG responses in normal rabbits remain scarce, complicating the establishment of normative ERG values. It is challenging to define the normal range of ERG values, given that the results of ERG tests can be influenced by a variety of factors. Efforts have been made to establish a standardized protocol for ERG in rabbits, aiming to enhance inter-laboratory comparison capabilities and promote conformity of methodologies for ERG data collection^7^. However, intrinsic factors, such as age can also influence the ERG response in humans^8^ and animals^9^.

In studies on neonatal rabbits^10^the observation of the a-wave occurs during the second week postpartum, followed by the emergence of the b-wave and oscillatory potentials at 2 to 3 weeks. The peak time and amplitude undergo dynamic changes until 5 weeks of age, which is the endpoint of the experiments. Additionally, a separate study compared 3-month-old mixed strain rabbits with their 1- to 2-year-old counterparts^7^. The findings revealed significantly lower b-wave amplitudes in young rabbits compared to older ones, with no significant difference in implicit time values. These investigations suggest age-related variations in normal ERGs among rabbits. Therefore, in experiments involving ERGs conducted both pre- and post-treatment administration, it is crucial to consider the normal variability over time as rabbits age, as this variability could influence results and complicate data interpretation, especially in long-term studies. However, the lack of research specifically investigating age-related changes in normal ERGs in adult rabbits remains a gap in the literature.

Furthermore, pigmentary status presents another variable affecting ERG outcomes, as evidenced by differing responses observed in albino and pigmented rabbits^11^. Those studies indicated that albino rabbits exhibit greater a- and b-wave amplitudes and shorter latency times. This can be partially ascribed to the augmented light exposure of receptor cells in albino rabbits due to the absence of melanin.

To the best of our knowledge, no investigation has been undertaken to compare age-related functional variations in albino and pigmented rabbits through ERG. Therefore, this study was designed to investigate the impact of natural aging on ERG waveforms in both albino and pigmented rabbits. Slit lamp ophthalmologic examination and imaging monitoring were utilized before ERG experiments in order to ensure normal eye physiology that would not affect the ERG signals. The dynamic changes of ERG data were assessed longitudinally over a period of 24 months using ff-ERG equipment.

## Results

### Ophthalmologic examination

Prior to conducting in vivo experiments with rabbits, we conducted comprehensive ophthalmologic evaluations using a slit lamp biomicroscope to assess their eye condition. This technique enables detailed visualization of anterior segment structures, including the adnexa, eyelids, conjunctiva, cornea, iris, lens, and anterior chamber, as well as posterior segment structures such as the vitreous, retinal vessels, optic nerve head, and retina. All eight rabbits were found to have clear corneas, normal iris and lens morphology, and no signs of inflammation or vascular abnormalities. These assessments ensured that only rabbits with clinically normal eyes were included in the study, minimizing confounding effects on ERG measurements.

### In vivo comparison retinal structure of white new Zealand (WNZ) and Dutch belted (DB) rabbits

**Fig. 1:**
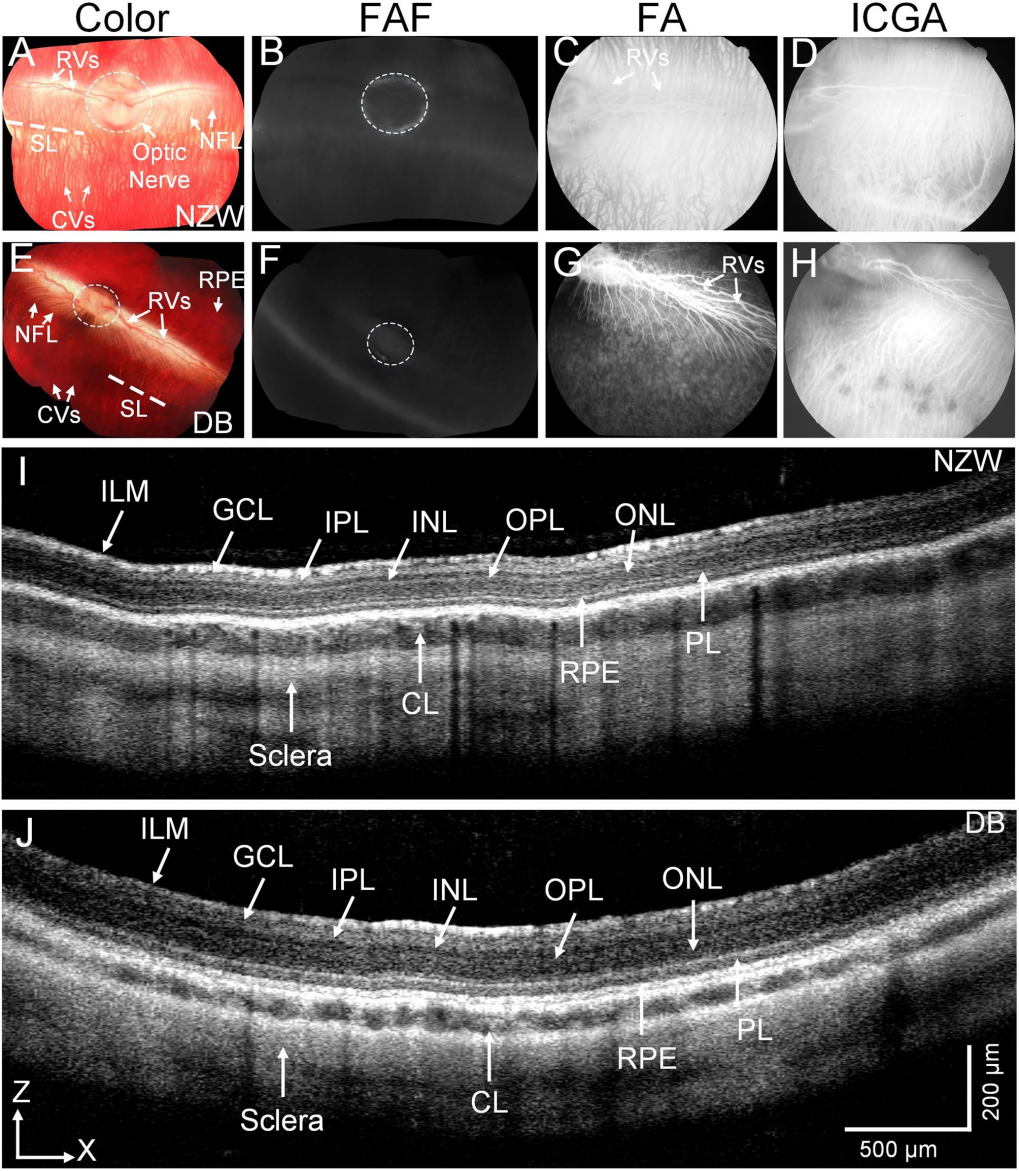
Multimodal imaging of New Zealand White (NZW) and Dutch-belted (DB) Rabbit retina:** (A** and **E**) Color fundus photography image of NZW (**A**) and DB (**E**) rabbits, respectively. These images clearly show morphology of retinal vessels (RVs), nerve fiber layer (NFL), choroidal vessels (CVs), retinal pigment epithelium (RPE), and optic nerve (white dotted circles). **(B** and **F**) Fundus autofluorescence images showing healthy retina without any evidence of RPE atrophy. **(C** and **G**) Fluorescein angiography of NZW and DB, respectively. (**D** and H) Indocyanine green angiography images clearly show structure of the choroidal vessels. (**I** and **J**) B-scan spectral domain OCT (SD-OCT) images demonstrating the cross section retina constructed from different layers such as inner limiting membrane (ILM), ganglion cell layer (GCL), inner plexiform layer (IPL), inner nuclear layer (INL), outer plexiform layer (OPL), outer nuclear layer (ONL), photoreceptor layer (PL), choroid layer (CL), and sclera. The OCT scan region of interest is indicated by scanning lines (SL) and marks as red dotted lines overlay on the fundus photographs (a and d), corresponding to the rabbit’s visual streak. This region was chosen for its high photoreceptor density and relevance to retinal function as a structure akin to the human macula.

Before testing the retinal function of the NZW and DB rabbits, we examined the retinal architectures of these rabbit strains using different imaging modalities including color fundus, fundus autofluorescence (FAF), and high-resolution spectral domain optical coherence tomography (SD-OCT) imaging (Fig. 1). The color fundus photography demonstrates normal retinal architecture with clear morphology of retinal vessels (RVs), choroidal vessels (CVs), nerve fiber layer (NFL), and optic nerve on both NZW (Fig. 1A) and DB (Fig. 1E) rabbits. The retinal pigment epithelium (RPE) layer was more clearly observed in the DB (Fig. 1E) as opposed to the NZW rabbits (Fig. 1A). The FAF images show no evidence of RPE atrophy, confirming the health of the retina (Fig. 1B, and F). Fluorescein angiography (FA) and indocyanine green angiography (ICGA) were performed at all imaging timepoints to assess retinal and choroidal vascular integrity. These angiographic assessments revealed no significant abnormalities or perfusion deficits in either New Zealand White or Dutch-Belted rabbits throughout the 24-month study period. Given the absence of detectable vascular changes, detailed FA and ICGA data are not shown but a representative image of each is shown in Fig. 1C–D and G–H. Instead, retinal and choroidal vasculature are described based on color fundus photography, which provided sufficient visualization for the purposes of this study.

High-resolution spectral domain optical coherence tomography (SD-OCT) images demonstrate the normal architecture of the retina with distinct structure including inner limiting membrane (ILM), ganglion cell layer (GCL), inner plexiform layer (IPL), inner nuclear layer (INL), outer plexiform layer (OPL), outer nuclear layer (ONL), photoreceptor layer (PL), choroid layer (CL), and sclera (Fig. 1I and J). These results confirmed no structural abnormalities that could affect functional testing. It is important to consider whether structural changes within the visual streak (VS), which holds a higher density of cone photoreceptors, could contribute to the cone pathway deficits observed in albino NZW rabbits. In this study, spectral-domain OCT imaging was focused on the VS region to assess retinal morphology longitudinally. Quantitative analysis of total retinal thickness in the central retina showed no statistically significant differences across 2, 7, 15, and 24 months in either strain (NZW: *p* = 0.6; DB: *p* = 0.5; one-way ANOVA). These results indicate stable retinal structure over time, supporting that the age-related decline in ERG responses, especially in the albino NZW group, is not associated with overt structural thinning, but may instead reflect functional or cellular changes.

### In vivo retinal function comparison between NZW and DB rabbits

**Fig. 2:**
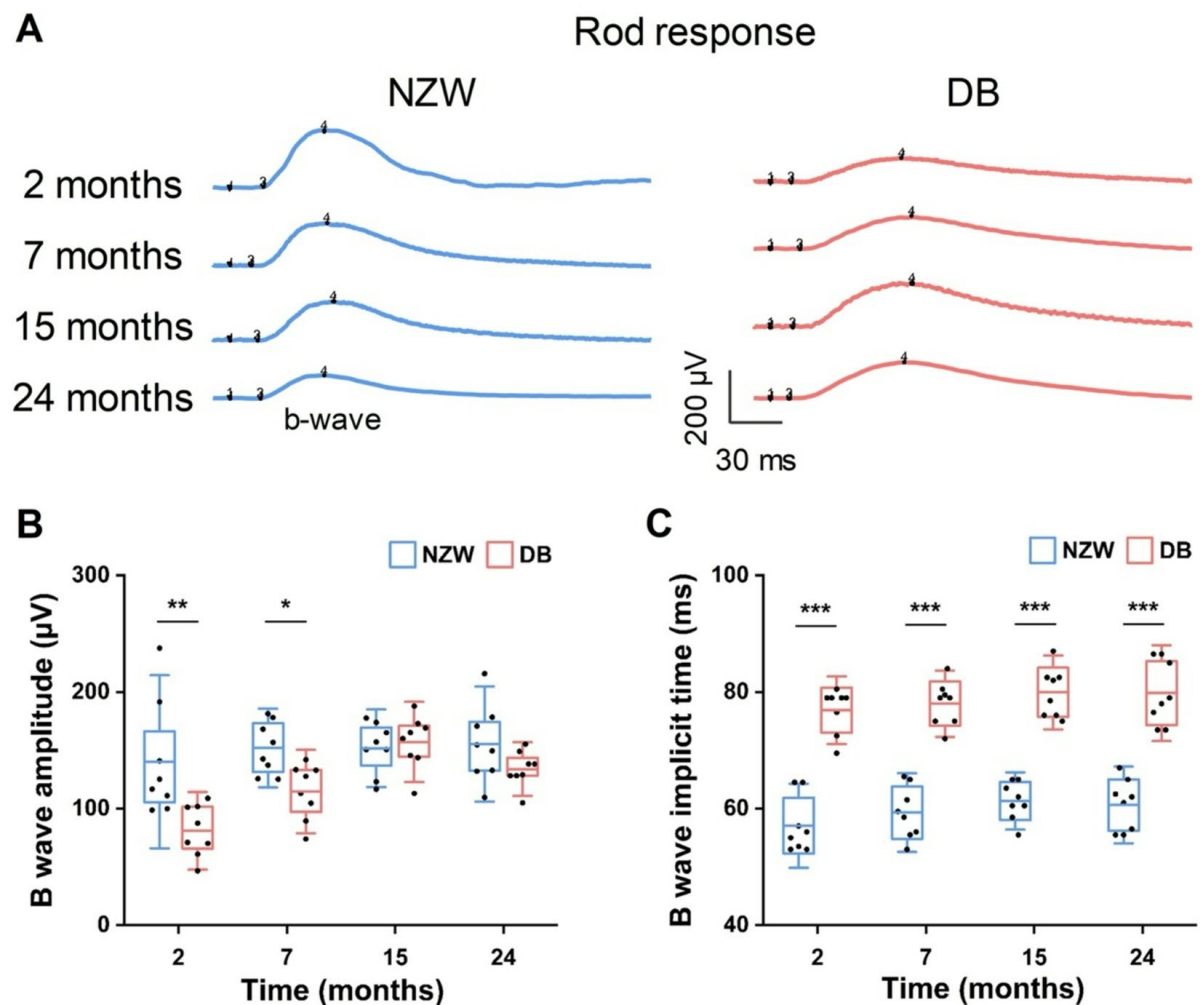
Comparison of rod responses in NZW and DB rabbits at different ages: (**A**) Representative rod ERG waveforms recorded to a scotopic 0.01 cd·s/m², flash under dark-adapted conditions in NZW and DB rabbits from 2 to 24 months of age. Scale bars = 30 ms (x-axis) and 200 µV (y-axis). (**B**) Raw B-wave amplitude and (**C**) implicit time of rod responses in NZW and DB rabbits over time. Horizontal bars = mean values; boxes = interquartile range; capped lines = standard deviation (*n* = 8, * *P* < 0.05, ** *P* < 0.01, *** *P* < 0.001).

**Fig. 3:**
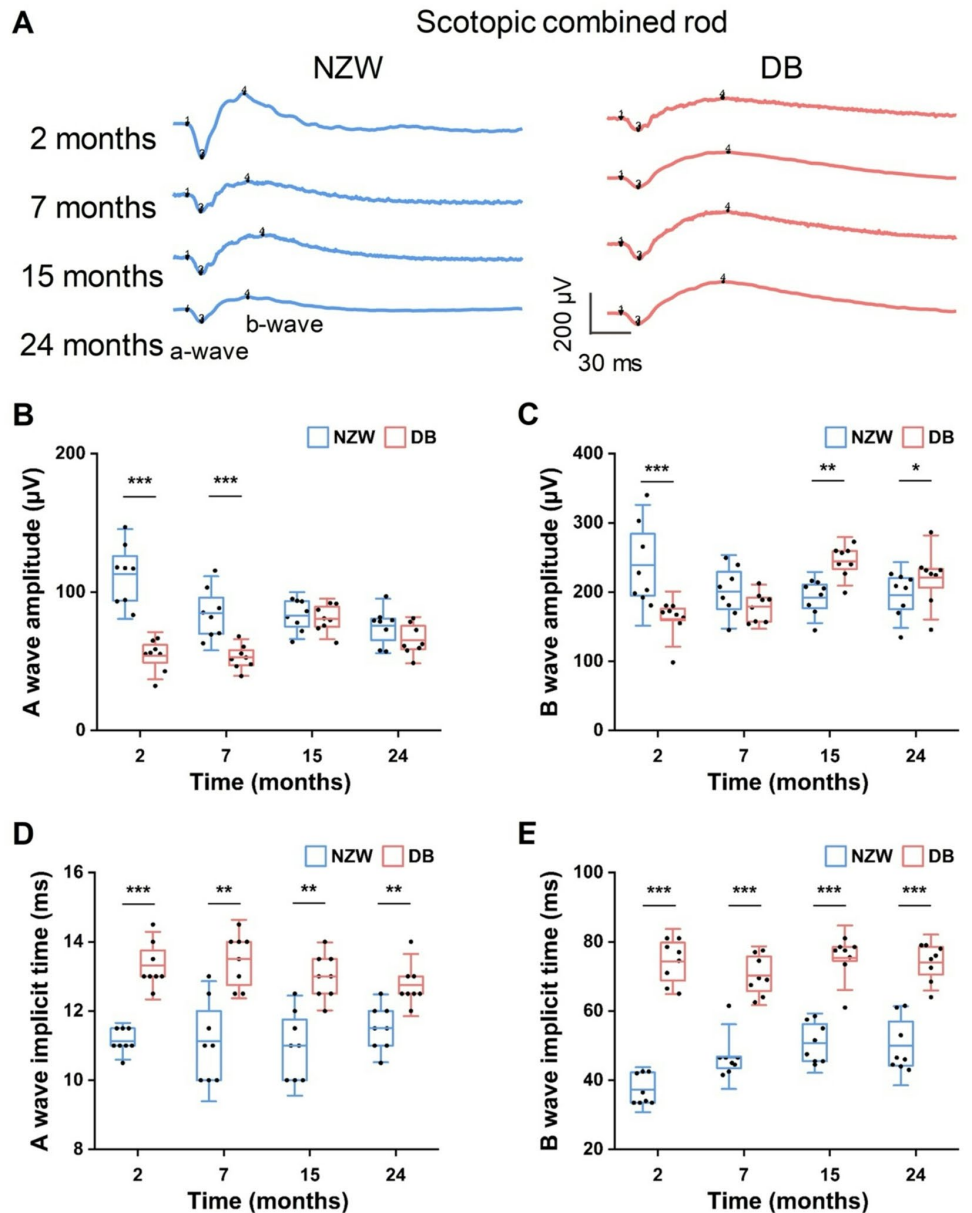
Comparison of combined rod and cone responses in NZW and DB rabbits at different ages: (**A**) Representative combined rod and cone ERG waveforms recorded to a scotopic 3.0 cd·s/m², flash under dark-adapted conditions in NZW and DB rabbits from 2 to 24 months of age. Scale bars = 30 ms (x-axis) and 200 µV (y-axis). (**B**) Raw A-wave amplitude, (**C**) B-wave amplitude, (**D**) A-wave implicit time, and (**E**) B-wave implicit time of scotopic combined rod and cone responses in NZW and DB rabbits over time. Horizontal bars = mean values; boxes = interquartile range; capped lines = standard deviation (*n* = 8, *P < 0.05, **P < 0.01, ***P < 0.001).

**Fig. 4:**
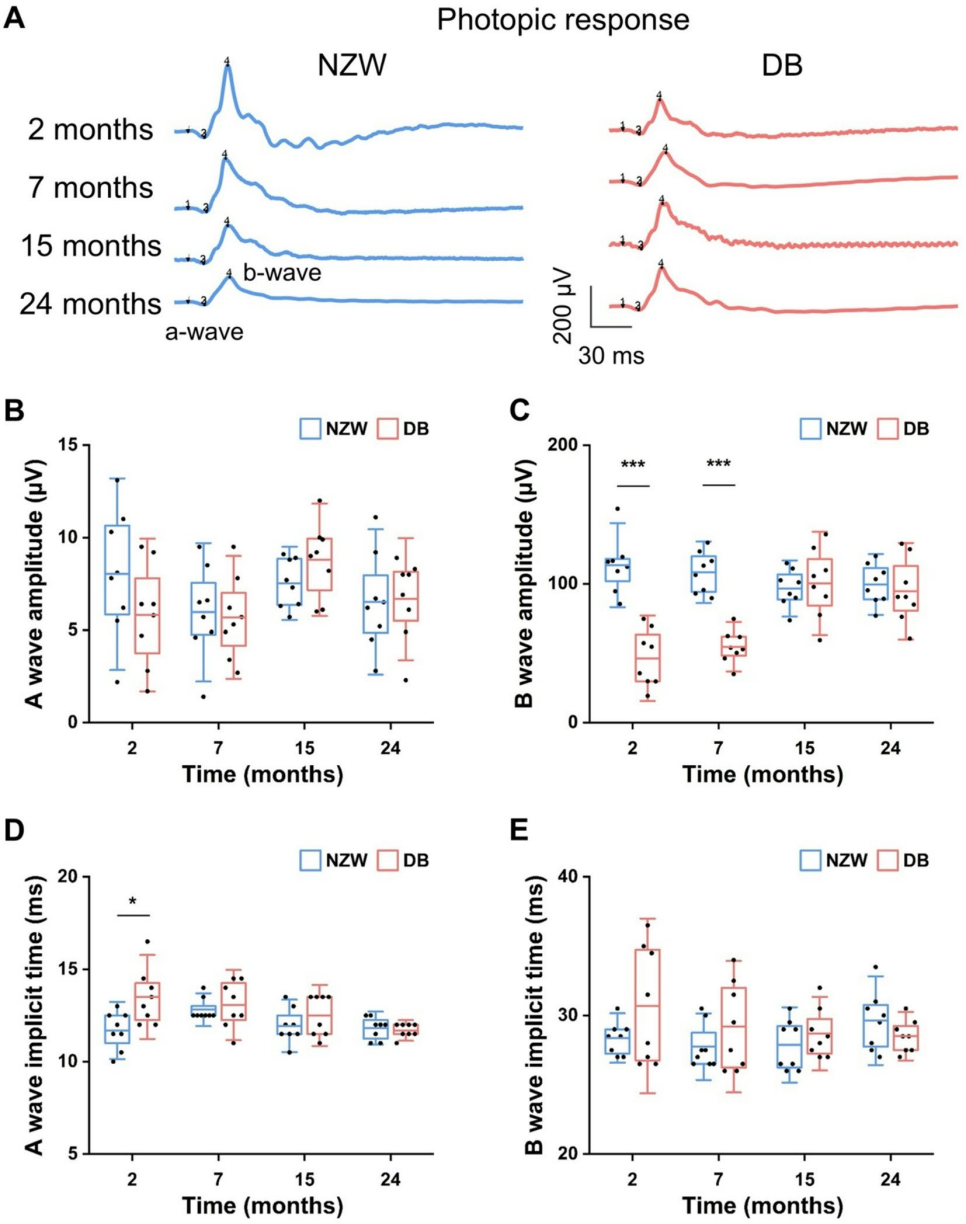
Comparison of photopic responses in NZW and DB rabbits at different ages: (**A**) Representative ERG waveforms recorded to a photopic 3.0 cd·s/m², flash under light-adapted conditions in NZW and DB rabbits from 2 to 24 months of age. Scale bars = 30 ms (x-axis) and 200 µV (y-axis). (**B**) Raw A-wave amplitude, (**C**) B-wave amplitude, (**D**) A-wave implicit time, and (E) B-wave implicit time of photopic responses in NZW and DB rabbits over time. Horizontal bars = mean values; boxes = interquartile range; capped lines = standard deviation (*n* = 8, *P < 0.05, ***P < 0.001).

**Fig. 5:**
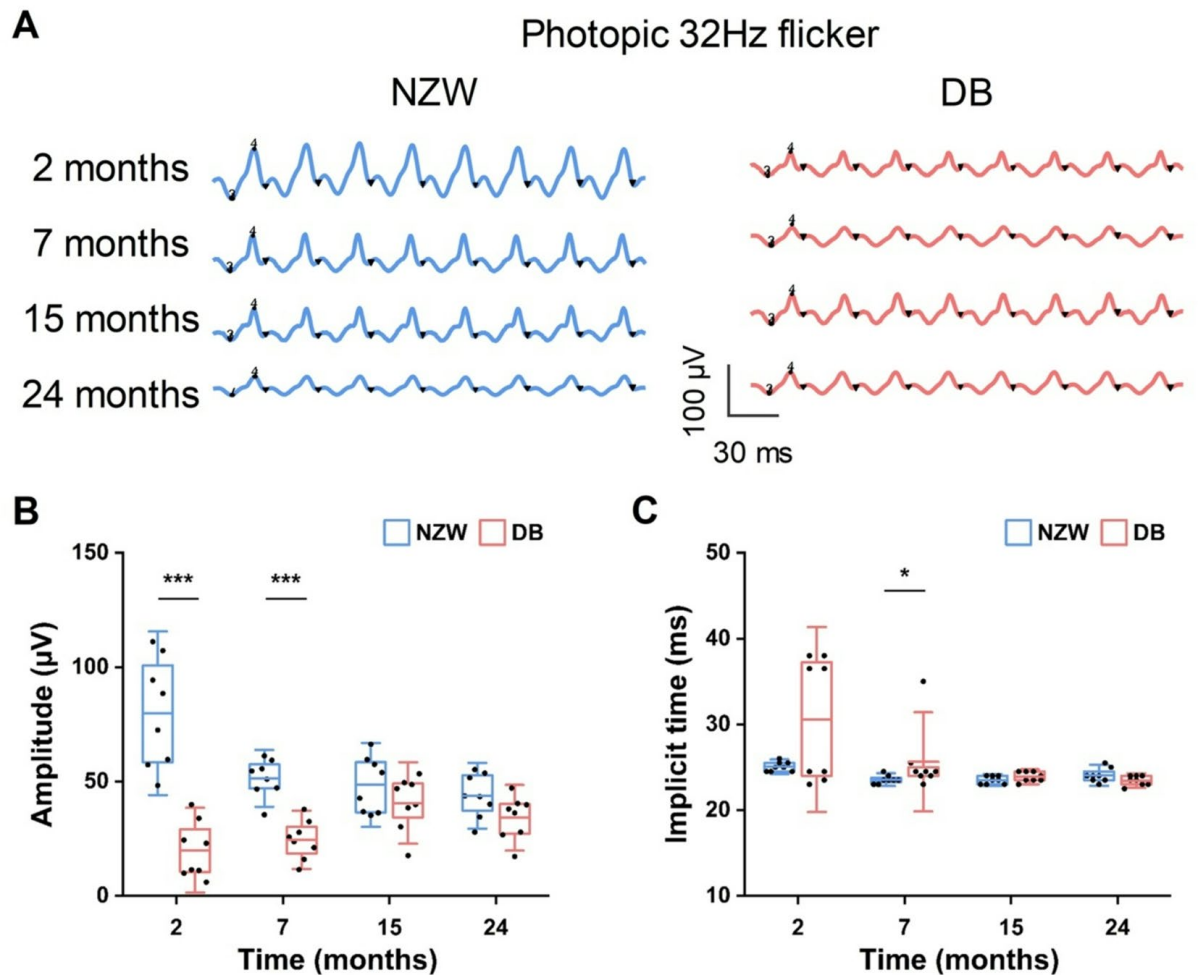
Comparison of cone responses in NZW and DB rabbits at different ages: (**A**) Representative ERG waveforms recorded to a photopic 3.0 cd·s/m², 30 Hz flash under light-adapted conditions in NZW and DB rabbits from 2 to 24 months of age. Scale bars = 30 ms (x-axis) and 100 µV (y-axis). (**B**) Raw amplitude and (**C**) implicit time of cone responses in NZW and DB rabbits over time. A significant difference between strains was observed only at 7 months (*P* < 0.05). Horizontal bars = mean values; boxes = interquartile range; capped lines = standard deviation (*n* = 8, * *P* < 0.05, **P < 0.01, ***P < 0.001).

To gain a better understanding of the differences in retinal function between NZW and DB rabbits, ERG was conducted to measure the electrical activity of the retina in response to a light stimulus. Briefly, the amplitudes and implicit times of both rabbits at four different time points (2, 7, 15, and 24 months) under scotopic and photopic conditions were recorded. All recorded data were subjected to analysis and displayed in four categories: rod response (scotopic 0.01 cd.s/m^2^, Fig. 2 and **Fig. S1**), combined rod and cone response (scotopic 3.0 cd·s/m², Fig. 3 and **Fig. S2**), cone response (photopic 3.0 cd·s/m², Fig. 4 and **Fig. S3**, and photopic 30 Hz flicker, Fig. 5 and **Fig. S4**) in order to facilitate a comparison of the retinal function. In general, NZW rabbits exhibited higher ERG amplitudes and shorter implicit times during early time points, particularly under scotopic conditions. These amplitudes declined slightly over time. In contrast, DB rabbits displayed lower initial amplitudes but demonstrated progressive increases as they aged.

In the rod response (Fig. 2), the b-wave amplitude of the NZW rabbits had significantly higher value relative to the DB at 2 and 7 months. The value was observed to be up to 73% higher (at 2 months; *P* < 0.01) and 33% higher (at 7 months; *P* < 0.05 vs. DB). Over time, the amplitudes became comparable, and no statistically significant difference was observed between the NZW and DB rabbits at 15 and 24 months (Fig. 2B). The b-wave implicit time was found to be approximately 25% shorter in the NZW (59.6 ± 4.4 ms) than the DB (78.7 ± 4.4 ms; *P* < 0.001) during all experimental periods (Fig. 2C).

Figure 3 depicts the response to a flash stimulus that elicits a rapid corneal negative potential, termed the a-wave, and a subsequent positive b-wave under dark-adapted conditions. The a-wave amplitude of the scotopic combined rod and cone response exhibited a similar tendency to that of the b-wave amplitude of the rod response, with notable discrepancies initially and then becoming comparable. At the initial two time points, the a-wave amplitude of the NZW rabbits was 109% (2 months) and 60% (7 months) higher than that of the DB rabbits, respectively (*P* < 0.001; Fig. 3B). The b-wave amplitude of the NZW rabbits also demonstrated a substantial difference at 2 months, reaching 239 ± 58 µV (148% of the DB; *P* < 0.001). However, the DB rabbits showed higher b-wave amplitudes at 15 months (127%; *P* < 0.01) and 24 months (113%; *P* < 0.05 vs. NZW; Fig. 3C). In terms of implicit time, the NZW rabbits are apparently faster than the DB rabbits in all cases. The a-wave is up to 18% faster and the b-wave is up to 50% faster in implicit time (*P* < 0.01 and *P* < 0.001 vs. DB; Fig. 3D and E).

The cone response was observed under light-adapted conditions, where the cone pathway can be assessed by suppressing the rod response in a bright background. Figure 4 shows an a-wave and a b-wave generated by cone photoreceptors, as well as a combination of ON and OFF bipolar cells, respectively. Unlike the combined scotopic rod and cone response, no significant differences were seen in a-wave amplitudes across time points (Fig. 4B). However, NZW rabbits had significantly greater b-wave amplitudes at 2 and 7 months (245% and 198%, respectively; *P* < 0.001; Fig. 4C). The a-wave implicit time in the NZW rabbits was 13% shorter than in the DB rabbits at only two months of age (*P* < 0.05), and no statistical differences were observed in the implicit times between the two groups in any of the other time points (Fig. 4D and E).

Figure 5 shows the ffERG elicited by a 30-Hz flicker stimulus. Rod photoreceptors are unable to follow fast 30-Hz flicker, so the results of the 30-Hz flicker are indicative of cone pathway function. Each stimulus flash in the flicker produces a response with a peak and a trough, where the amplitude of the flicker is defined as the trough-to-peak amplitude, and the implicit time of the flicker response is defined as the time between the stimulus flash and the corresponding response peak. At 2 and 7 months, NZW rabbits exhibited significantly larger flicker response amplitudes compared to DB rabbits, approximately fourfold and twofold greater, respectively (*P* < 0.001; Fig. 5B). However, the significance observed at 7 months in implicit time (Fig. 5C) was partially influenced by a single low-amplitude outlier in the DB group. Reanalysis excluding this data point reduced the group difference but maintained statistical significance, suggesting this timepoint should be interpreted with caution. Over the 24-month observation period, NZW rabbits displayed a gradual decline in flicker amplitude, while DB rabbits showed a slight increase, resulting in comparable amplitudes between strains at 15 and 24 months. Implicit times were significantly shorter in NZW rabbits (23.6 ± 0.5 ms) than in DB rabbits (25.6 ± 3.9 ms) at 7 months (*P* < 0.05; Fig. 5C), but no significant differences were observed at other timepoints.

To evaluate the potential influence of interocular variability on ERG outcomes, paired statistical comparisons between left and right eyes were performed for each ERG parameter and timepoint. Across the cohort, no significant interocular differences were detected (*p* > 0.05), supporting the validity of using either the average or a randomly selected eye per animal. Notably, in one DB rabbit at 7 months, a significant interocular discrepancy in 30 Hz flicker amplitude was observed, coinciding with the outlier noted in Fig. 5C. This finding reinforces the importance of inter-eye comparison as a quality control step in longitudinal ERG studies. Accordingly, for this animal and timepoint, only the higher-quality eye recording was retained for analysis.

In summary, the results demonstrated that NZW rabbits exhibit robust retinal electrical responses in the early stages and rapid scotopic responses with short implicit times at 2-, 7-, 15-, and 24-months, in comparison to DB rabbits. DB rabbits exhibited a natural and gradual enhancement in retinal function over the period from 2 to 24 months.

To assess both the effect of age (time points) and rabbit strain on ERG amplitude responses, we performed a two-way repeated measures ANOVA using OriginPro 9.0 as shown in Table 1. The analysis included time (2, 7, 15, and 24 months) as a within-subject factor and strain (NZW vs. DB) as a between-subject factor. Post-hoc pairwise comparisons were conducted using Bonferroni correction to adjust for multiple testing. For NZW rabbits, significant age-related differences were observed primarily at earlier time points in the scotopic 0.01 cd.s/m² a-wave amplitudes and 30 Hz flicker responses. Specifically, a significant decline in scotopic a-wave amplitude was seen between 2 months and 7 months (*p* = 6.86 × 10⁻⁴), and further decreases were evident between 2 months and 15 months (*p* = 2.86 × 10⁻⁴) and 2 months and 24 months (*p* < 0.0001). In the 30 Hz flicker response, significant differences were observed between 2 and 7 months (*p* = 4.85 × 10⁻⁴), 2 and 15 months (*p* < 0.0001), and 2 and 24 months (*p* < 0.0001). No statistically significant changes were detected among the later time points (7, 15, and 24 months). In contrast, DB rabbits exhibited progressive functional enhancement across multiple parameters up to 15 months of age, followed by a modest decline thereafter. Significant changes between 2 and 15 months were observed in several parameters, including increases in scotopic b-wave (*p* < 0.0001), scotopic a-wave (*p* = 0.0018), photopic b-wave (*p* = 1.99 × 10⁻⁴), and 30 Hz flicker amplitude (*p* = 0.02992). Additional differences were found between 2 and 24 months and between 7 and 15 months in both scotopic and photopic responses. The photopic response b-wave amplitude, distinct from the photopic b-wave, was particularly sensitive, showing consistent and significant changes across multiple time point comparisons (e.g., 7 m vs. 15 m: *p* < 0.0001). These results highlight differential aging patterns in retinal function between NZW and DB rabbit strains.

**Table 1:** Two-Way repeated measures ANOVA with bonferroni Post-hoc Test.

Time comparison	Strain	Scotopic 0.01 cd.s/m^2^ b-wave	Scotopic 3.0 cd.s/m^2^ a-wave	Scotopic 3.0 cd.s/m^2^ b-wave	Photopic 3.0 cd.s/m^2^ a-wave	Photopic 3.0 cd.s/m^2^ b-wave	Photopic response b-wave amplitude
2 m vs. 7 m	NZW	1	**0.00868**	1	0.87718	1	0.07287
2 m vs. 15 m	NZW	1	**0.004496**	1	1	1	**0.03347**
2 m vs. 24 m	NZW	1	**4.13E-04**	1	1	1	**0.00911**
7 m vs. 15 m	NZW	1	1	1	1	1	1
7 m vs. 24 m	NZW	1	1	1	1	1	1
15 m vs. 24 m	NZW	1	1	1	1	1	1
2 m vs. 7 m	DB	0.87584	1	1	1	1	1
2 m vs. 15 m	DB	**0.00139**	**0.0163**	**0.0206**	0.5151	**0.005**	**0.58574**
2 m vs. 24 m	DB	**0.04861**	1	0.2505	1	**0.0149**	1
7 m vs. 15 m	DB	0.23752	**0.0105**	0.1431	0.4096	**0.0251**	1
7 m vs. 24 m	DB	1	1	1	1	0.0751	1
15 m vs. 24 m	DB	1	0.7622	1	1	1	1

Bold values indicate statistically significant comparisons (*p* < 0.05) after Bonferroni correction.

## Discussion

In this study, we longitudinally evaluated retinal function in albino New Zealand White (NZW) and pigmented Dutch Belted (DB) rabbits from 2 to 24 months of age using full-field electroretinography (ff-ERG)^12^. Our results revealed distinct age-related functional changes in these strains, highlighting the influence of pigmentation and aging on ERG parameters. The findings have implications for model selection in preclinical retinal research.

NZW rabbits consistently demonstrated larger ERG amplitudes and shorter implicit times than DB rabbits across multiple stimulus conditions, particularly at younger ages (2 and 7 months). These findings are consistent with previous reports comparing ERG responses in albino versus pigmented rabbits^13–15^. The enhanced responses in albino animals likely reflect increased light availability at the photoreceptor level due to reduced melanin-mediated light absorption and increased intraocular scatter^13,16^. This is supported by the understanding that melanin plays a crucial role in modulating light absorption, reducing intraocular stray light, and protecting retinal tissue^17,18^. Interestingly, some pigmented rodents exhibit greater ERG amplitudes than albinos^19–21^ indicating that melanin’s influence is context-dependent and complex.

A likely explanation for the higher amplitudes and shorter implicit times in the scotopic ERG responses of albino NZW rabbits is the increased effective light stimulus at the photoreceptor level. Although both strains received the same external light stimulus, the number of photons reaching the photoreceptors is likely greater in albino rabbits due to reduced melanin-mediated absorption in the retinal pigment epithelium (RPE) and increased intraocular light scatter. This elevated photon availability enhances the retinal light stimulus, leading to stronger a- and b-wave amplitudes and faster implicit times under scotopic conditions. A similar trend was observed under photopic conditions, though statistical significance was not achieved, likely due to the inherently lower signal-to-noise ratio and greater variability of photopic ERG signals. Together, the altered effective stimulus and potential physiological differences in photoreceptor function likely contribute to the observed ERG differences, with the former playing a dominant role.

The difference in amplitude and implicit time between albino and pigmented rabbits was most pronounced under scotopic conditions. Rod photoreceptors, which dominate scotopic vision, are highly sensitive to light^22^. In the absence of melanin, albino rabbits may experience greater effective light stimulation, accelerating phototransduction and leading to stronger and faster ERG responses under dim lighting. Conversely, under photopic conditions, mediated by cone photoreceptors, the difference in implicit time between NZW and DB rabbits was minimal. This suggests that melanin has a lesser role in cone-mediated function during bright-light adaptation.

While melanin’s primary function is light absorption, our findings and prior studies suggest its role in retinal physiology is more complex^17,18^. These mixed results point to a multifactorial influence of melanin, potentially involving its roles in retinal development, antioxidant defense, and structural maintenance.

Age-related changes in ERG responses were observed in both strains but followed distinct trajectories. NZW rabbits exhibited a gradual decline in cone and postreceptoral function between 2 and 24 months, while their rod-specific responses were relatively preserved. In contrast, DB rabbits showed an initial increase in ERG amplitude, peaking at 15 months, followed by a modest decline. These patterns may reflect differences in maturation timelines, consistent with reported differences in lifespan and development between rabbit strains^23^. The delayed peak function in DB rabbits may indicate a slower developmental course compared to NZW rabbits.

We also observed that the most significant functional differences between the strains occurred during early adulthood (2–7 months), suggesting that pigmentation-related factors and developmental changes have a more pronounced impact on retinal function during this period. Notably, while NZW rabbits displayed higher amplitudes overall, the degree of age-related decline was also more substantial, particularly in cone-driven responses.

The relatively stable rod responses in NZW rabbits despite overall functional decline may relate to their inherently lower rod photoreceptor density and reduced baseline function in albino animals. This limited functional reserve may lead to smaller measurable changes over time, creating the appearance of stability. However, this should not be interpreted as increased resilience to aging, but rather as a limitation in dynamic range. Previous studies in albino animals have documented anatomical differences in rod system development. For example, Grant and Jeffrey et al. reported that the absence of melanin in the RPE of albino mammals leads to a reduction in rod photoreceptor numbers and early-onset structural defects in the retina^24,25^. Similarly, Naash et al. found that albino mice exhibited more severe photoreceptor degeneration and greater ERG deficits when carrying a mutant opsin gene compared to pigmented^26^. Furthermore, Iwai-Takekoshi et al. showed that albino mouse retinas have compromised RPE morphology and disrupted intercellular junctions, further contributing to functional deficits^27^.

To ensure accurate interpretation of these findings, we controlled for several potential confounding variables, including interocular variability and light exposure. Paired-eye comparisons showed no significant differences in ERG parameters across most timepoints, validating the use of either averaged or randomly selected eyes per animal. The exception, a single DB rabbit with asymmetric flicker response at 7 months, was treated as an outlier, demonstrating the importance of interocular comparison as a quality control step.

Technical factors such as anesthesia depth, electrode positioning, and body temperature are known to influence ERG outcomes^7,28,29^. In our study, we standardized all experimental conditions, including anesthetic protocol and testing time, to minimize variability. Despite this, some inter-animal differences persisted, as commonly reported^29,30^. This suggests a need for robust statistical methods and intraindividual comparisons in longitudinal ERG studies.

Beyond congenital pigmentation differences, the functional consequences of melanin loss may extend to acquired depigmenting conditions, such as ocular vitiligo. Although typically patchy and localized, these conditions may similarly compromise retinal support and light regulation. Thus, our findings may inform future studies exploring functional outcomes in acquired pigment disorders.

Finally, aging independently contributes to retinal decline. Age-related changes, including photoreceptor degeneration, RPE dysfunction, and oxidative stress, impair light responsiveness and reduce ERG amplitudes while prolonging implicit times^31,32^. In this context, it is crucial to age-match animal groups and consider the aging trajectory of each strain when interpreting ERG data.

In conclusion, this study provides insights into the long-term differences in retinal function between NZW and DB rabbits, shedding light on age-related changes and disparities between albino and pigmented rabbits. Through a comprehensive ERG analysis conducted over four time points spanning from 2 to 24 months, significant functional variations were observed between the two rabbit strains. These findings collectively elucidate the intricate interplay between retinal function, pigment presence, and age-related changes in rabbit models, offering valuable insights into the underlying mechanisms of vision and highlighting the potential implications for understanding retinal disorders and age-related changes in rabbit models as well as emphasize the importance of selecting an appropriate for preclinical studies.

## Materials and methods

### Animal preparation

The research ensured adherence to the ARRIVE (Animal Research: Reporting of In Vivo Experiments) guidelines and the ARVO (The Association for Research in Vision and Ophthalmology) Statement for the Use of Laboratory Animals in Ophthalmic and Vision Research. All experimental procedures followed relevant guidelines, regulations, and ethical standards. Approval for the research protocol (Protocol PRO00010388) was obtained from the Institutional Animal Care & Use Committee (IACUC) of the University of Michigan before commencing the study.

For this research, eight rabbits comprising four New Zealand White and four Dutch Belted of varying genders, aged between 2.1 and 24 months, and weighing 1.65 to 4.21 kg, were specifically bred at the Center for Advanced Models and Translational Sciences and Therapeutics (CAMTraST) within the University of Michigan Medical School. Rabbits were chosen for their eye similarities to humans, including axial length and anatomy, making them suitable for ophthalmic investigations. These rabbits were housed in a controlled environment with regulated temperature and lighting, following a 12-hour light-dark cycle to mimic a standard diurnal rhythm. The animal room was illuminated with an average light intensity of 61.08 lx at the top cages, 33.93 lx in the middle cages, and 21.11 lx near the floor cages. To minimize the influence of differential light exposure, cage positions were rotated approximately every 2–4 weeks, ensuring that no animal was continuously housed in a specific lighting zone. Additionally, pigmented (Dutch-Belted) and albino (New Zealand White) rabbits were initially and subsequently distributed evenly across the different cage levels during each rotation to avoid strain-dependent bias in light exposure. The animals had continuous access to water and received standard laboratory food to fulfill their nutritional needs.

Before any procedures or imaging, anesthesia was administered to all rabbits via intramuscular injections. Ketamine (40 mg/kg, 100 mg/mL) sourced from JHP Pharmaceuticals (Rochester, MI, USA) and xylazine (5 mg/kg, 100 mg/mL) from Anased^®^ (Boise, ID, USA) were utilized for induction. Pupillary dilation, essential for ophthalmic examinations, was achieved by administering tropicamide 1% ophthalmic and phenylephrine hydrochloride 2.5% ophthalmic solutions. Additionally, topical anesthesia, using 0.5% tetracaine or proparacaine, was applied to ensure the rabbits’ comfort during the procedures. To maintain corneal hydration throughout the experiments, phosphate-buffered saline (BRL, Life Technologies; Grand Island, NY, USA) was regularly administered, typically every minute. During all anesthesia procedures, the rabbits’ vital signs including mucous membrane color, heart rate, respiratory rate, oxygen saturation, and body temperature were continuously monitored. Body temperature was measured using a rectal thermometer (Medline, MDS9952BZ, CA, USA) and maintained within the normal physiological range (~ 37 °C) using a circulating warm water heating pad (Gaymar Stryker^®^ TP650 Pump, NY, USA). The duration of anesthesia for each procedure was approximately 2 h following anesthetic injection. A pulse oximeter (V8400D Capnograph & SpO2 Digital Pulse Oximetry, Smiths Medical, MN, USA) was utilized to assess oxygen saturation and monitor the rabbits’ respiratory status.

### Timepoint selection

The timepoints of 2, 7, 15, and 24 months were selected to represent distinct stages in the rabbit lifespan relevant to retinal development and aging. At 2 months, rabbits are considered young adults with mature retinal function. The 7-month timepoint corresponds to full young adult maturity, while 15 months reflects mid-adulthood. The 24-month timepoint represents a more mature stage, as rabbits generally have a lifespan of 5 to 8 years. These intervals enabled longitudinal assessment of retinal function across the aging process, allowing us to capture both early and late age-related changes in ERG parameters.

### Ophthalmologic examination

Ophthalmologic assessments were conducted both prior to imaging and at regular intervals thereafter. These evaluations included a comprehensive examination of the adnexa, eyelids, conjunctiva, cornea, anterior chamber, iris, lens, vitreous, optic nerve, retina, and retinal vessels utilizing slit lamp bio-microscopy (SL120, Carl Zeiss, Germany). Following pupil dilation, subsequent examination of the vitreous, optic nerve, and retina was performed using a contact fundus lens (Volk Optical Inc, Mentor, OH, USA).

### Imaging procedure

Ophthalmologic assessments were initiated when the animals were 2 months old and continued until 24 months of age. Imaging examinations, including fundus photography, fundus autofluorescence (FAF), fluorescein angiography (FA), and indocyanine green angiography (ICGA), and OCT, were performed at 2, 7, 15, and 24 months to longitudinally monitor age-related retinal changes. The color fundus photography, FAF, FA, and ICGA, were employed to evaluate retinal characteristics in both WNZ and DB rabbits. Imaging procedures commenced with color fundus imaging, followed by sequential assessments using FAF, FA, and ICGA. Subsequent to ICGA imaging, optical coherence tomography (OCT) was utilized. To facilitate imaging, a pediatric Barraquer wire speculum was utilized to maintain eyelid openness. Fundus photography was conducted utilizing the Topcon 50 EX system (TRC 50EX, Topcon Corporation, Tokyo, Japan) to visualize the posterior eye segment.

For FA imaging, a 0.20 mL injection of fluorescein sodium solution (Akorn, Lake Forest, IL, USA) was administered intravenously through the marginal ear vein of the anesthetized rabbit. FA images were captured immediately post-injection and at one-minute intervals for 5 to 15 min, allowing monitoring of fluorescence intensity and vascular changes. Similarly, ICGA imaging involved the injection of 0.20 mL of indocyanine green (ICG) dye (HUB Pharmaceuticals LLC, Patheon, Italy). ICGA contrast was imaged at the same intervals as FA to assess vascular perfusion and potential abnormalities.

Optical coherence tomography (OCT) imaging was conducted utilizing a Ganymede-II-HR OCT system (Thorlabs, Newton, NJ) with adjustments as detailed previously^33–35^. In summary, two superluminescent light-emitting diodes emitting at a central wavelength of 905 nm were employed to illuminate the corneal surface, with the resultant light focused on the fundus via rabbit eye optics. The average power of the OCT probing light was maintained at 0.8 mW. The lateral and axial resolutions were measured at 3.8 μm and 4.0 μm, respectively, with the system capable of achieving an imaging depth of 1.9 mm. Rabbits were positioned on a customized platform, and eye alignment was optimized under the ophthalmic lens using a CCD camera to ensure accurate visualization of the region of interest. B-scan images were obtained with a resolution of 512 × 1024 A-lines and a sampling rate of 36 kHz. The OCT system facilitated cross-sectional imaging of the retina, offering detailed structural information at high resolution.

In vivo OCT imaging was consistently performed centered on the rabbit’s visual streak, a horizontal band of higher photoreceptor density that serves as the functional equivalent of the human macula. To improve spatial reference, the corresponding location of the OCT scan area is marked on the fundus images in the Results section (Fig. 1). This ensured that structural assessments focused on the most functionally relevant retinal region.

### Electroretinography (ERG)

Full-field electroretinography (ff-ERG) was employed on both NZW and DB rabbits as previous study^36^. Briefly, all animals used for ERG experiments were kept in a dark room for an hour. After dark adaptation, the rabbits were anesthetized (approximately 10–15 min before electrode application), and topical anesthesia was applied. ERG testing was consistently performed between 9:00 AM and 11:00 AM to reduce variability due to circadian rhythms in retinal function.

ERG-Jet contact lens electrodes (The Electrode Store, Enumclaw, WA, USA) were utilized following corneal hydration with a 2.5% hypromellose ophthalmic solution (Akorn Inc, Lake Forest, IL, USA). Subcutaneous placement of reference electrodes and a ground electrode (needle electrodes, The Electrode Store, Enumclaw, WA, USA) behind the bilateral ears and in the scruff, respectively, was conducted. All procedural steps were carried out under dim red-light conditions.

ERGs were acquired utilizing a Ganzfeld configuration with the LKC UTAS 3000 electrophysiology system (LKC Technologies, Gaithersburg, MD, USA). ERG recordings were performed under both scotopic and photopic conditions. During data acquisition, signals were amplified at a gain of 2500× and bandpass filtered between 0.312 Hz and 500 Hz. The digitization rate was set at 2000 Hz to ensure high-fidelity recording of retinal responses.

Scotopic ERGs were obtained at flash intensities of 0.01 cd.s/m^2^ for isolating rod responses and 3.0 cd.s/m^2^ for combined rod-cone responses. Each flash had a duration of 4 ms and was delivered at a rate of 1 Hz with a 1-second interstimulus interval. Five to ten responses were averaged per stimulus condition. The flash stimulus intensities were initially calibrated using a calibrated photometer and were routinely verified at regular intervals during the study. No significant changes in stimulus intensity were observed, and calibration logs were maintained throughout the experimental period.

Photopic ERGs were recorded after 10 min of light adaptation to a white background light of 32 cd/m² to suppress rod activity. The test flash intensity was 3.0 cd·s/m². The same 4 ms flash duration, and 1 Hz frequency were used, with averaging of 5–10 responses per condition.

Photopic flicker ERGs were recorded at 30 Hz following the light adaptation period using the same 3.0 cd·s/m² flash intensity. Individual flicker waveforms were not averaged across flashes, but 5–10 repeated recordings were acquired and averaged to improve signal-to-noise ratio. Waveform consistency across trials was confirmed within each subject.

ERG analysis included measurements of a-wave amplitude and implicit time (from pre-stimulus baseline to the trough), and b-wave amplitude and implicit time (from trough to peak). A xenon white flash was used as the stimulus source.

During post-acquisition processing, the recorded data were resampled at 1000 Hz, and a 0.312–300 Hz bandpass filter was applied. Each recording included a 500 ms time window, consisting of a 10 ms pre-flash baseline and a 490 ms post-flash period. No notch filtering was applied during analysis. The slight adjustments in sampling frequency and filter bandwidth between acquisition and post-processing were made to optimize data handling and analysis, while preserving the integrity of the ERG waveform components.

ERG recordings were conducted separately from the imaging sessions to avoid potential anesthesia-related effects on retinal function. Specifically, ERG recordings were performed one week prior to each imaging session, and each procedure was conducted under a separate anesthesia. Origin 9 software (OriginLab Corporation, MA, USA) was used to plot a-wave and b-wave amplitudes, a-wave and b-wave implicit times as a function of times.

### OCT retinal thickness quantification

Retinal thickness was measured from the inner limiting membrane (ILM) to the retinal pigment epithelium (RPE) using the built-in caliper tool in the OCT software. For each eye, three measurements were taken at adjacent points in the central retina and averaged to obtain a representative thickness value. Statistical comparisons of retinal thickness across age groups were performed using one-way ANOVA followed by Tukey’s post hoc test. A p-value < 0.05 was considered statistically significant.

### Statistical methods

All statistical analyses were performed using OriginPro 9.0 software (OriginLab Corporation, MA, USA). Prior to any statistical testing, the normality of each dataset was assessed using the Shapiro–Wilk test. Parametric tests (e.g., one-way or two-way repeated measures ANOVA) were used when the data followed a normal distribution (*p* > 0.05), and non-parametric alternatives (e.g., Wilcoxon signed-rank test) were applied when normality was not met. To evaluate the effects of rabbit strain and age (time) on retinal electrophysiological responses, we employed a two-way repeated measures ANOVA, with strain (NZW vs. DB) as a between-subject factor and time (2, 7, 15, and 24 months) as a within-subject factor. Post-hoc pairwise comparisons were conducted using Bonferroni correction to adjust for multiple testing. To address concerns about statistical independence, we evaluated interocular differences at each timepoint using paired t-tests. If no significant differences were detected between eyes across timepoints or ERG conditions (*p* > 0.05), final analyses used the average of both eyes per rabbit.

## Data Availability

The corresponding authors can provide the data supporting the plots and other findings of this study upon request.
